# RNA interference in the cat flea, *Ctenocephalides felis*: Approaches for sustained gene knockdown and evidence of involvement of *Dicer-2* and *Argonaute2*^☆^

**DOI:** 10.1016/j.ijpara.2018.04.006

**Published:** 2018-11

**Authors:** Catriona H. Edwards, John Baird, Erich Zinser, Debra J. Woods, Sophie Shaw, Ewan M. Campbell, Alan S. Bowman

**Affiliations:** a Institute of Biological and Environmental Sciences, School of Biological Sciences, University of Aberdeen, Tillydrone Avenue, Aberdeen AB24 2TZ, UK; b Zoetis Inc, 333 Portage Street, Kalamazoo, Michigan 49007, USA; c Centre for Genome Enabled Biology and Medicine, University of Aberdeen, 23 St. Machar Drive, Old Aberdeen AB24 3RY, UK

**Keywords:** RNA interference, *Ctenocephalides felis*, *GST*, Dicer-2, Argonaute2, Nucleases, Artificial feeding, Knockdown

## Abstract

Effective RNA interference (RNAi) methods have been developed in many pest species, enabling exploration of gene function. Until now RNAi had not been attempted in the cat flea, *Ctenocephalides felis*, although the development of RNAi approaches would open up potential avenues for control of this important pest. This study aimed to establish if an RNAi response occurs in adult *C. felis* upon exposure to double-stranded RNA (dsRNA), which administration methods for dsRNA delivery could bring about effective gene knockdown and to investigate dynamics of any RNAi response. Knockdown of 80% of *GSTσ* was achieved by intrahaemoceolic microinjection of ds*GSTσ* but this invasive technique was associated with relatively high mortality rates. Immersing *C. felis* in ds*GSTσ* or ds*Dicer-2* overnight resulted in 65% knockdown of *GSTσ* or 60% of *Dicer-2*, respectively, and the degree of knockdown was not improved by increasing the dsRNA concentration in the bathing solution. Unexpectedly, the greatest degree of knockdown was achieved with the continuous administration of dsRNA in whole blood via a membrane feeding system, resulting in 96% knockdown of *GSTσ* within 2 days and sustained up to, at least, 7 days. Thus, unlike in many other species, the gut nucleases do not impair the RNAi response to ingested dsRNA in *C. felis*. A modest, but significant, upregulation of *Dicer-2* and *Argonaute2* was detectable 3 h after exposure to exogenous dsRNA, implicating the short-interfering RNA pathway. To our knowledge this study represents the first demonstration of experimentally induced RNAi in the cat flea as well as giving insight into how the gene knockdown response progresses.

## Highlights

• First known demonstration of gene knockdown in a flea, the cat flea (*Ctenocephalides felis*)
• Strong, transient gene knockdown by immersion in double-stranded (ds) RNA solution.
• Strong, sustained gene knockdown by continuous feeding of dsRNA in blood.
• dsRNA treatment induces an increase in *Dicer-2* and *Argonaute-2* gene expression.
• The knockdown approach is useful for pesticide and pathogen transmission studies in fleas.

## Graphical abstract

**Figure:**
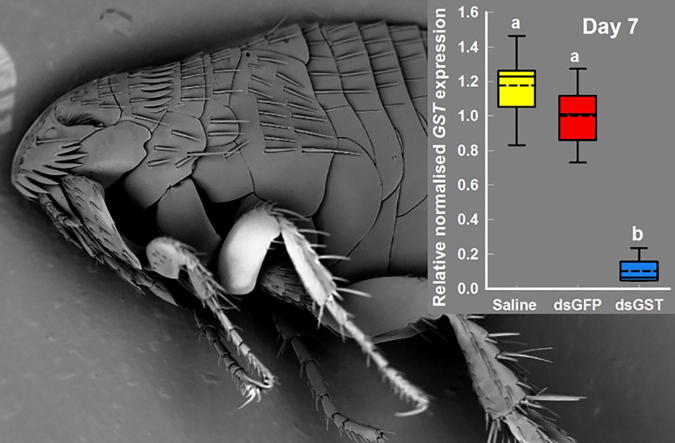

## 1 Introduction

The cat flea, *Ctenocephalides felis*, is a major pest species of companion animals worldwide. Across Europe a range of flea infestations have been reported, for example surveys found 14.3% of cats in Germany (Beck et al., 2006), 22.9% of cats in Hungary (Farkas et al., 2009), and 21% of cats in the UK (Bond et al., 2007) had fleas, with the vast majority identified as *C. felis*. Cat fleas are also commonly the dominant flea species found on dogs, for example 88% of fleas infesting dogs in Spain (Gracia et al., 2008) and 61% of fleas recovered from dogs in Georgia in the USA (Durden et al., 2005) were *C. felis.* Cat flea infestations cause irritation and discomfort, and can trigger a severe allergic reaction known as flea allergy dermatitis (FAD). FAD can cause intense discomfort, pruritus, self-mutilation and anaemia in extreme cases, making prompt treatment and elimination of fleas important to relieve symptoms and prevent recurrence (Carlotti and Jacobs, 2000). *Ctenocephalides felis* is a vector of several infectious agents, including *Bartonella henselae* (Chomel and Kasten, 2010) and *Rickettsia felis* (feline rickettsiae) and acts as the intermediate host of the dog tapeworm *Dipylidium caninum* (Beugnet and Marié, 2009). Due to the abundance of cat flea infestations and associated issues, there is a constant demand for flea treatments, which account for a large portion of the US $3.4 billion spent on ectoparasiticides for companion animals annually (Woods and Knauer, 2010). Alongside concerns about the potential for cat fleas to become resistant to existing products, this large commercial market is a major driving force for the development of new flea control products (Rust, 2016).

RNA interference (RNAi) is a functional genomics approach which is useful for discovery and validation of novel insecticide targets, as well as for functional genetic research in disease vectors such as the cat flea. Gene knockdown, which is based on a naturally occurring antiviral defence mechanism, is achieved by administration of exogenous long double-stranded RNA (dsRNA), which is initially cleaved into ≈21–23 nucleotide pieces termed short-interfering RNAs (siRNAs) by a cytoplasmic ribonuclease III, Dicer-2. siRNAs are incorporated into the RNA-induced silencing complex (RISC), where they bind to complementary target RNA, which is then cleaved by the enzyme Argonaute2 (AGO2). Cleavage occurs whether or not the dsRNA is derived from a natural viral infection or generated in vitro, to target a gene transcript in gene knockdown experiments. RNAi has become a widely used entomological tool to investigate gene function, select and validate pesticide targets, and is even being developed for use directly as a pest control method (Huvenne and Smagghe, 2010, Scott et al., 2013, Zotti et al., 2018). However, to our knowledge the method has thus far not been attempted in *C. felis.*

While RNAi can be an effective tool in many insect species, knockdown efficiency is highly variable between species. Some insects are highly susceptible to RNAi, mounting a strong systemic response to even small amounts of dsRNA, while others are much less sensitive and large amounts of dsRNA may be needed to elicit even a minor knockdown response (Zotti and Smagghe, 2015). In particular coleopterans have been shown to be highly sensitive to dsRNA, with long-lasting strong knockdown occurring in a number of species including *Tribolium castaneum* (Bucher et al., 2002, Tomoyasu and Denell, 2004), *Diabrotica virgifera virgifera* and *Leptinotarsa decemlineata* (Baum et al., 2007). In contrast, many Lepidoptera mount an RNAi response much less efficiently (Terenius et al., 2011). The route by which dsRNA is administered can also have an impact on the outcome, with some species such as the migratory locust, *Locusta migratoria*, able to mount a strong response to dsRNA delivered via injection, but negligible via feeding (Luo et al., 2013). Possible reasons for the variability between and within species are deficiencies in RNAi machinery expression or activity (Bellés, 2010, Swevers et al., 2011), limited uptake mechanisms, or degradation of dsRNA, especially within the gut lumen (Wynant et al., 2012, Christiaens et al., 2014, Ren et al., 2014, Wang et al., 2016). As RNAi has not previously been reported in *C. felis* or any others of the Order Siphonaptera, it was not known where on this spectrum of RNAi responses *C. felis* would fall or indeed if RNAi could be detected in *C. felis* at all.

The aim of the present study was to establish whether *C. felis* was able to mount an RNAi response to exogenous dsRNA and demonstrate robust knockdown of the targets *glutathione S-transferase* (*GSTσ*) and *Dicer-2*. Further, if successful gene knockdown was achieved, the aim was to assess the efficiency and ease of use of different dsRNA administration methods. Finally, the involvement of the siRNAi pathway was examined by investigating the upregulation of two ribonucleases, *Dicer-2* and *AGO2*, in response to exogenous dsRNA. This study is, to our knowledge, the first to demonstrate gene knockdown by RNAi in the cat flea, and thus opens new avenues for research in this common pest species.

## 2 Materials and methods

### 2.1 Flea specimens

For microinjection and assessment of associated mortality, adult *C*. *felis* were collected from an artificially reared colony at the University of Aberdeen, derived from an artificially reared colony provided by Zoetis Inc. (MI, USA). For subsequent trials (excluding feeding trials) *C. felis* were from eggs supplied by Elward II Labs (CA, USA), derived from an artificially reared cat flea colony. All life stages were kept in an insectary at ≈26 °C, ≈80% relative humidity and 12:12 light:dark (L:D) cycles. Following emergence, adults were fed sheep blood in acid-citrate-dextrose (ACD) (4 parts sheep blood: 1 part ACD) (TCS Biosciences, UK) maintained at 37 °C via a Hemotek feeding system. Three times per week the adult rearing chambers were cleaned out, eggs and frass collected, and fresh sheep blood in ACD supplied. Eggs and frass were transferred to larval rearing pots containing fine sand and larval rearing media (74% finely ground dog food, 25% dried Brewer’s yeast and 1% dried porcine blood). Larval rearing pots were left undisturbed until adults emerged (≈21 days).

The feeding trial was conducted at Zoetis Inc., Kalamazoo, USA with *C. felis* eggs supplied by Elward II Labs (CA, USA), and reared in larval rearing media consisting of 74% finely ground laboratory canine diet, 25% dried Brewer’s yeast and 1% dried bovine blood, and fine sand, under insectary conditions similar to those described above.

### 2.2 Target sequence identification

A sigma class *GSTσ*, a detoxification enzyme, was selected as a target for gene knockdown trials. The *Drosophila melanogaster* GST S1 (transcript variant A, NM_166216.2) protein sequence, retrieved from GenBank, was used as a query sequence to search against publically available *C. felis* expressed sequence tags (ESTs) and transcriptome shotgun assembly databases (NCBI) using tBLASTn (Camacho et al., 2009). The identity of the top C. *felis GSTσ* (GR301946.1) hit was confirmed by tBLASTx against the NCBI non-redundant database. Only regions of the *C. felis* sequence with identity to the *D. melanogaster* sequences were used for subsequent primer design.

Sequences for *Dicer-2* and *AGO2* were identified in a newly assembled in-house *C. felis* transcriptome using BLASTx, *E*-value cut off 1e^−20^, searching with protein sequences retrieved from GenBank (*Bombyx mori Dicer-2* (AB566386.1); *B. mori AGO2* (NM_001043530.2)). Identity of selected hits was confirmed by BLASTx against the NCBI non-redundant database and manual checking. The *Dicer-2* and *AGO2* sequences used for subsequent primer design are presented in Supplementary Data S1. Both sequences are also available in GenBank (*Dicer-2*, MH170281; and *AGO2*, MH170282).

### 2.3 Primer design and testing

Primers were designed using Primer3Plus (http://www.bioinformatics.nl/cgi-bin/primer3plus/primer3plus.cgi/) and checked for self-complementarity using OligoCalc (http://www.basic.northwestern.edu/biotools/oligocalc.htmL) (Table 1). Primers for quantitative PCR (qPCR) were designed implementing qPCR settings on Primer3Plus. For bacterial production of dsRNA, the potential amplicons were checked for internal restriction sites using NEBcutter 2 (Vincze et al., 2003) and restriction sites added at the 5′ end of primer sequences (Table 1).

**Table 1:** Sequences of oligonucleotides used in this study for both double-stranded RNA (dsRNA) cloning and quantitative PCR (qPCR). *Bgl*II (AGATCT) and *Xho*I (CTCGAG) restriction sites are indicated by underlined bold text.

Target/Purpose	Primer name	Oligonucleotide sequence	Expected product size (bp)
*GSTσ* (dsRNA)	Bgl*GSTσ*A F	AGTCAG**AGATCT**TATTGGAGGTCGACGGAAAG	348
	Xho*GSTσ*A R	CTAGGA**CTCGAG**GATAACTCCGGCGAAAACAA	
*GSTσ* (qPCR)	qG*STσ*A F	TGAGAACAACATGCCTACTTACAAA	112
	q*GSTσ*A R	CACTCGGACATCTTCGAATTTT	
*GSTσ* sequencing	*GSTσ*A F	TATTGGAGGTCGACGGAAAG	n/a
*Dicer-2* (dsRNA)	ds*Dicer-2* F	CAGTTC**CTCGAG**ATCGCAATGCTGATGGTACA	470
	ds*Dicer-2* R	GCAGC**AGATCT**CAGCACAAGGAGCAAATTCA	
*Dicer-2* (qPCR)	q*Dicer-2* F	CATCTCATGGAAAGCGAAGC	91
	q*Dicer-2* R	AAGTTGCGTAACCCGGTATG	
*AGO2* qPCR	q*AGO2* F	CAATATGGGGGCAGAGTTTC	143
	q*AGO2* R	ATGGCCCACACACGTATTTC	
Reference genes	*Ef* F	TCGTACTGGCAAATCCACAG	145
	*Ef* R	CATGTCACGGACAACGAAAC	
	*GAPDH* F	ACCCAAAAGACTGTGGATGG	117
	*GAPDH* R	CGGAATGACTTTGCCTACAG	

Primers were tested by PCR to confirm production of a single product of the correct size. Reaction mixes consisted of 25 µL of Biomix (Bioline, UK), 22 µL of H_2_O, 1 µL of 10 µM forward and reverse primer mix and 2 µL of cDNA (1/2 or 1/5 dilution of mixed *C. felis* cDNA) and were run with the following conditions: 94 °C 5 min, (94 °C 30 s, 58 °C 45 s, 72 °C 45 s) x35 cycles, 72 °C 10 min. PCR products were electrophoresed in a 2% agarose Tris-borate-EDTA (TBE) gel with SYBR safe DNA gel stain (Invitrogen by Thermo Fisher Scientific, USA) alongside a 100 bp DNA ladder (Bioline) to confirm amplicon size.

Reverse transcription-qPCR (RT-qPCR) primers were tested as follows: *GSTσ* primers – 10 µL of iTaq Sybr Green Supermix (Bio-Rad), 5 µL of H_2_O, 1 µL of 10 µM primer mix, 4 µL of template (serial 10-fold dilutions of standard mixed life stage *C. felis* cDNA); *Dicer-2* and *AGO2* primers, 5 µL of iTaq Sybr Green Supermix (Bio-Rad), 0.5 µL of H_2_O, 0.5 µL of 10 µM primer mix, 4 µL of mixed *C. felis* cDNA (10-fold serial dilutions). A CFX96 Real-Time PCR Detection system (Bio-Rad Laboratories, USA) was used to run the RT-qPCRs with the following conditions: 95 °C 3 min, (94 °C 10 s, 58 °C 30 s) ×40 cycles, melt-curve 65–95 °C rising in 0.5 °C increments. All primer tests were conducted in duplicate. Primer efficiency and melt curves were analysed with CFX Manager software (version 3.1) (Bio-Rad), to ensure a single peak was present for each primer set.

### 2.4 Cloning into bacteria

Insert sequences (*GSTσ* or *Dicer-2*) for cloning were amplified using primers with restriction enzyme sites (Table 1). PCR mix consisted of 25 µL of Biomix Red (Bioline), 23 µL of H_2_O, 1 µL of 10 µM primer mix, 2 µL of mixed standard *C. felis* cDNA and the reaction run with the following conditions: 94 °C 5 min, (94 °C 30 s, 58 °C 45 s, 72 °C 45 s) ×35 cycles, 72 °C 15 min. PCR product size (10 µL) was confirmed by electrophoresis in a 2% agarose TBE gel. PCR products were purified using a QIAgen PCR purification kit (QIAgen, UK), according to manufacturer’s instructions. Four 40 µL reactions were pooled at this stage, and eluted with 40 µL of H_2_O. The purified PCR product was quantified using a Nanodrop 1000 spectrophotometer (Agilent, UK).

*Escherichia coli* DH5α cells (Promega, UK) containing empty pL4440 plasmid (Addgene, USA) were grown overnight in 5 mL of Luria-Bertani (LB) broth with 5 µL of 100 mg/mL ampicillin. Plasmid was extracted from 3 mL of the overnight culture using a QIAgen Spin MiniPrep kit, according to manufacturer’s instructions. Plasmid DNA was eluted with 50 µL of H_2_O and quantified using a Nanodrop 1000 spectrophotometer.

Plasmid (1 µg) 1 µg or insert DNA was restriction enzyme digested with *Bgl*II and *Xho*I restriction enzymes (Promega). Three restriction digest reactions were pooled and products purified using the QIAgen PCR purification kit according to the manufacturer’s instructions. Products were eluted with 20 µL of H_2_O and quantified by a Nanodrop 1000 spectrophotometer and stored at −20 °C until use.

Insert (*GSTσ* or *Dicer-2*) and pL4440 DNA were ligated together by incubating 4 µL of digested insert DNA (≈400 ng) with 1 µL of digested pL4440 (≈80 ng) and 5 µL of Instant sticky-end ligase master mix (New England Biolabs, USA) at room temperature for 5 min. Ligation mixes were then chilled on ice for ≈15 min before transformation into *E. coli* JM109 (Promega). Ligation mix (1 µL) was added to 100 µL of just-thawed JM109 cells and the transformation procedure carried out according to the manufacturer’s instructions, and transformants grown overnight at 37 °C on LB ampicillin (0.1 mg/mL) agar. Colony PCR and restriction enzyme digests of positive colonies were performed to confirm the presence of the correct insert sequence.

Plasmids containing the correct insert were used to transform *E. coli* HT115 cells (Caenorhabditis Genetics Center (CGC), University of Minnesota, USA), using standard bacterial cloning procedures. Transformed HT115 cells were grown overnight at 37 °C on LB ampicillin (0.1 mg/mL) tetracycline (0.01 mg/mL) plates. Picks of resultant colonies were used to spike 5 mL of LB with ampicillin (0.1 mg/mL) and tetracycline (0.01 mg/mL), and glycerol stocks made from overnight cultures. Stocks were stored at −80 °C for future use. Plasmids were extracted from transformed HT115 cell cultures using the QIAgen Miniprep Spin kit, according to the manufacturer’s instructions, and sent to Eurofins Genomics (Ebersberg, Germany) for dideoxy chain termination sequencing to confirm the presence of expected insert sequences. Sequencing of the *GSTσ*::L4440 construct used Cf*GSTσ*A primer (Table 1); standard vector primer M13 uni (−21) (TGTAAAACGACGGCCAGT) was used for sequencing the *Dicer-2*::L4440 construct. The sequences were aligned with the expected dsRNA sequences using ClustalW with default settings in MEGA 7 (Kumar et al., 2016).

### 2.5 Bacterial production of dsRNA

dsRNA production was produced by induction of a T7 promoter using isopropyl-b-d-thiogalactopyranoside (IPTG), as described in Timmons et al. (2001). In brief, HT115 pL4440-dsRNA cells (*GSTσ*::L4440, *Dicer-2*::L4440 constructs) described in Section 2.4, or *GFP*::L4440 (Plasmid #11335, Addgene, USA) were grown overnight in 5 mL of LB with 0.1 mg/mL of ampicillin and 0.01 mg/mL of tetracycline at 37 °C with shaking (200 rpm). Overnight cultures were diluted 1/100 in 2xYT broth (8 g of Tryptone (Sigma Aldrich), 5 g of Bacto Yeast Extract (Sigma Aldrich), 2.5 g of NaCl (Sigma Aldrich) per 500 mL of H_2_O) with 0.1 mg/mL of ampicillin and 0.01 mg/mL of tetracycline. Cultures (500 mL) were incubated at 37 °C with shaking (200 rpm), until reaching OD_595_ ≈0.4. IPTG was then added for a final concentration of 0.4 mM to induce expression of dsRNA and cultures incubated at 37 °C 200 rpm for a further 4 h. Cells were then pelleted by centrifugation at 600 *g* for 5 min at 4 °C, supernatant discarded and the pelleted cells stored at −80 °C until use.

For dsRNA extractions from cell pellets from 100 mL of cell culture, cells were resuspended in 5 mL of Tri Reagent (Sigma Aldrich), and extractions performed according to the manufacturer’s instructions. A chloroform wash was performed twice, and the solution incubated at 4 °C for 1 h following addition of 100% isopropanol to allow maximum RNA precipitation. The resulting RNA pellet was washed twice with 75% EtOH, air dried and resuspended in ≈300 µL of nuclease-free H_2_O before incubation at 55 °C for 10 min. RNA was quantified by a Nanodrop 1000 spectrophotometer. The presence of a dsRNA band of the correct size was confirmed by electrophoresis in a 2% agarose TBE gel. dsRNA was stored at −80 °C. Before use, NaCl solution was added to dsRNA solutions for a final concentration of 0.9% saline. The sizes of ds*GSTσ*, ds*Dicer-2* and ds*GFP* were 323 bp, 441 bp and 869 bp, respectively.

### 2.6 Administration of dsRNA to *C. felis* by intra-haemocoelic microinjection

Microinjection needles were made from 8.9 cm glass capillaries (#3-000-203-G/X, Drummond Scientific Company, USA) using a Narshige Scientific Instrument Lab (Japan) needle puller. Prior to injection, newly-emerged unfed adult *C. felis* were immobilised by chilling on ice for 5–10 min. For injections, fleas were placed on double-sided sticky tape, and individually secured with autoclave tape straps over the anterior end of their bodies. Fleas were injected with 69 nL of 1 µg/µL of ds*GSTσ* or ds*GFP* between body segments at the posterior end of the abdomen using a Nanoject II microinjection system (Drummond Scientific Company) and a Leica MZ95 dissecting microscope (Leica Biosystems, Germany). When injection was successful the abdomen could be seen to expand. Overtly injured or killed fleas were removed after injections. After injection, groups of approximately 10 fleas were transferred to 15 mL centrifuge tubes with filter paper platforms, and kept under insectary conditions (described in Section 2.1). Surviving fleas were collected 96 h post-treatment, chilled on ice, then pierced in the abdomen with a 23 gauge needle and placed in RNAlater (Ambion, Thermo Fisher Scientific, UK), up to 10 fleas in 500 µL, as per McIntosh et al. (2016). Samples were stored at −80 °C prior to processing.

### 2.7 Assessing mortality associated with microinjection and immersion treatments

To assess the impact of different delivery methods, newly emerged unfed *C. felis* were anaesthetised by placing on ice for 5–10 min. Approximately 60 fleas were then microinjected with 69 nL of 0.9% saline, as described in Section 2.6, and two groups of approximately 40 fleas soaked for 17 h in 0.9% saline or not treated. For soaking, groups of 3–4 fleas were soaked for 17 h at 4 °C in 50 µL of 0.9% saline. Following treatment, any obviously injured or dead individuals were removed and cat fleas were transferred to 15 mL centrifuge tubes, approximately 10 fleas per tube. Cat fleas were then transferred to insectary conditions (described in Section 2.1) and mortality monitored daily for 10 days post-treatment.

### 2.8 Administering dsRNA to *C. felis* by immersion

To assess the ability of ds*GSTσ* to knockdown its target by immersion in dsRNA solution, newly emerged *C. felis* adults were collected, chilled on ice and divided into treatment groups of approximately 10 fleas (four groups for immersion in ds*GSTσ*, three groups for each of the controls). Fleas were chilled on ice for 5–10 min then 3–4 fleas were transferred to Eppendorf tubes with 40 µL aliquots of 1.5 µg/µL of ds*GSTσ*, 1.5 µg/µL of ds*GFP* or 0.9% saline. After placement in solutions, fleas were incubated at 4 °C for ≈17 h. Fleas were then transferred in groups of 10–15 mL centrifuge tubes with a filter paper platform to discourage jumping, transferred to insectary conditions and samples collected 72 h later. Only surviving fleas were collected, as described in Section 2.7. Samples were stored at −80 °C. For the ds*Dicer-2* assessment, the soaking volume was 30 µL and the dsRNA concentration was 2 µg/µL of ds*Dicer-2* or ds*GFP* and a saline control was not included. Samples were collected 48 h after removal from soaking solutions. Six groups of ds*GFP* and four groups of ds*Dicer-2*-treated fleas were collected.

To assess how knockdown progressed over time, newly emerged adult fleas were soaked in 40 µL of 2 µg/µL of ds*GSTσ* or ds*GFP*, and otherwise handled as above (see Section 2.8). Surviving fleas were collected 24, 48, 72 and 96 h after removal from dsRNA solution, as above (see Section 2.6). To investigate whether dsRNA dosage had an effect on knockdown, newly-emerged adult fleas (four groups of 10 fleas per treatment) were soaked for ≈17 h in 40 µL of 2 µg/µL of ds*GFP*, 2 µg/µL of ds*GSTσ* or 4 µg/µLof ds*GSTσ*. Fleas were collected at 72 h as described in Section 2.6.

### 2.9 Administration of dsRNA to *C. felis* by feeding

Newly-emerged adult *C. felis* were divided into 24 groups of ≈100 fleas and placed into separate flea rearing chambers, with eight groups for each of the three treatments (ds*GSTσ*, ds*GFP* or 0.9% saline). The flea rearing chambers were kept in an “artificial dog” system, adapted from methods described by Wade and Georgi (1988), with flea chambers held at 25 °C, 75% relative humidity (RH) and blood heated to 37 °C in feeding vessels held above the flea chambers and sealed with Nescofilm membrane (Bando Chemical Ind. Ltd., Japan). Fleas were fed on citrated bovine blood for 5 days prior to introduction of dsRNA or saline.

For feeding with the experimental solutions, each group of approximately 100 fleas was fed with 2 mL of freshly prepared blood/test solution mix, consisting of 110 µL of 2 µg/µL of dsRNA or 110 µL of 0.9% saline mixed with 1890 µL of citrated bovine blood. At regular intervals (2, 4 and 7 days after introduction of test solutions to feeding media) the feeding chambers were cleaned, 15 individuals collected from each group, and remaining cat fleas placed back into feeding chambers and provided with fresh blood/test solution mix. Upon collection, fleas were pierced in the abdomen with a 23 gauge needle and 15 placed in 1 mL of RNAlater solution (Invitrogen by Thermo Fisher Scientific), kept at room temperature overnight and then stored at −80 °C for optimal RNA preservation as described by McIntosh et al. (2016). The feeding experiment was carried out at Zoetis Inc. (Kalamazoo, USA) and samples were shipped on dry ice to the University of Aberdeen, UK for sample processing and knockdown assessment.

### 2.10 Upregulation of siRNAi machinery genes

Newly emerged adult *C. felis* were collected, chilled on ice for approximately 5 min and assigned to groups of approximately 10 in 15 mL centrifuge tubes. Fleas were soaked overnight in 30 µL of 0.9% saline or 2 µg/µL of ds*GST*, as described in Section 2.8. After soaking, fleas were kept under insectary conditions (see Section 2.1) until collection 1, 2, 3, 6 or 24 h after removal from solution and collected, as described in Section 2.6. Four groups of 10 fleas were collected at each time point for each treatment.

### 2.11 RNA extraction and cDNA synthesis

RNA extractions were performed on groups of 4–5 fleas using a Zymo RNA Insect and Tissue Microprep kit (Zymo Research, USA). Fleas were removed from RNAlater solution and crushed in 100 µL of lysis buffer using micropestles before addition of 700 µL of lysis buffer and transferral to bashing bead tubes for further processing. Following transfer to the spin columns, the centrifugation steps were extended to 5 min to allow solutions to fully pass through columns. On-column DNase treatment was performed first, washing the column by centrifuging with 400 µL of RNA wash buffer for ≈1 min, then 3 µL of RQ1 DNase, 3 µL of 5× RQ1 buffer (Promega) and 24 µL of RNA wash buffer were added to each column and incubated for 15 min at 30 °C. Columns were centrifuged for 30 s at 15,347*g*. The remaining protocol was completed following DNase treatment, and RNA was eluted from columns by the addition of 8 µL of H_2_O, allowed to stand for 1 min, then centrifuged for 30 s at 15,347*g*. RNA was quantified by a Nanodrop 1000 spectrophotometer and stored at −80 °C

cDNA was synthesised with an iScript cDNA synthesis kit (Bio-Rad). The reaction mix consisted of: 2 µL of 5× reaction buffer, 0.5 µL of iScript reverse transcriptase, 200 ng of RNA, oligo(dT) and random hexamer primers, and nuclease-free H_2_O was added for a final volume of 10 µL. Reactions were incubated for 5 min at 25 °C, 30 min at 42 °C, and finally 5 min at 85 °C. cDNA was stored at −20 °C.

### 2.12 RT-qPCR

Gene knockdown was assessed by examination of gene expression by RT-qPCR of the target gene and reference genes. For ds*GSTσ* microinjection and soaking trials, the following reaction mix was used: 10 µL of iTaq universal Sybr Green supermix (Bio-Rad), 1 µL of 10 µM primers, 5 µL of H_2_O, 4 µL of 1/200 dilution cDNA (≈5 ng/µL). For the time-course and dosage experiments (ds*GSTσ*) the reaction mix consisted of: 5 µL of iTaq universal Sybr Green supermix, 0.5 µL of 10 µM primers, 0.5 µL of H_2_O and 4 µL of 1/200 dilution cDNA. For assessment of *Dicer-2* knockdown and upregulation of RNAi machinery, the reactions were as for the time-course experiments, but 1/50 dilution of sample cDNA was used as template (≈20 ng/µL). All samples were run in triplicate. In all cases, 10-fold serial dilutions of mixed *C. felis* cDNA were used to assess primer efficiency, and duplicate no template controls were included for each primer set. Reactions were run on a CFX96 Real-Time PCR Detection system (Bio-Rad) with the following conditions: 95 °C 3 min, (94 °C 10 s, 58 °C 30 s) ×40 cycles, melt-curve 65–95 °C rising in 0.5 °C increments. Primer efficiency and melt curves were analysed with CFX Manager software (version 3.1) (Bio-Rad).

### 2.13 Data analysis

In Microsoft Excel (2013), RT-qPCR data were used to calculate the relative quantity (R0) of each transcript measured in each sample. R0 was calculated using the equation R0 = 1/(1 + *E*)^Cq^, where *E* is the efficiency of the given primer set and Cq is the average crossing point for triplicate reactions of a given sample. The R0 of the gene of interest was normalised by dividing by the geometric mean of the R0s of two reference genes, *Ef1α* and *GAPDH* (McIntosh et al., 2016). For two treatment comparisons, the statistical significance of results was assessed via a two-sample *t*-test, assuming equal variance. In trials involving more than two treatments, the overall treatment effect was assessed by one-way ANOVA, and multiple comparisons then performed by Tukey’s test if a statistically significant (*P* < 0.05) treatment effect had been determined. Statistical analyses were performed with Minitab version 18. Kaplan-Meier was used for survival curve assessment, with a log-rank (Mantel-Cox) test to compare the survival curves. Kaplan-Meier was performed using GraphPad Prism v. 5.04.

## 3 Results

### 3.1 Microinjection of dsRNA can cause significant knockdown of a target gene in *C. felis*

**Fig. 1:**
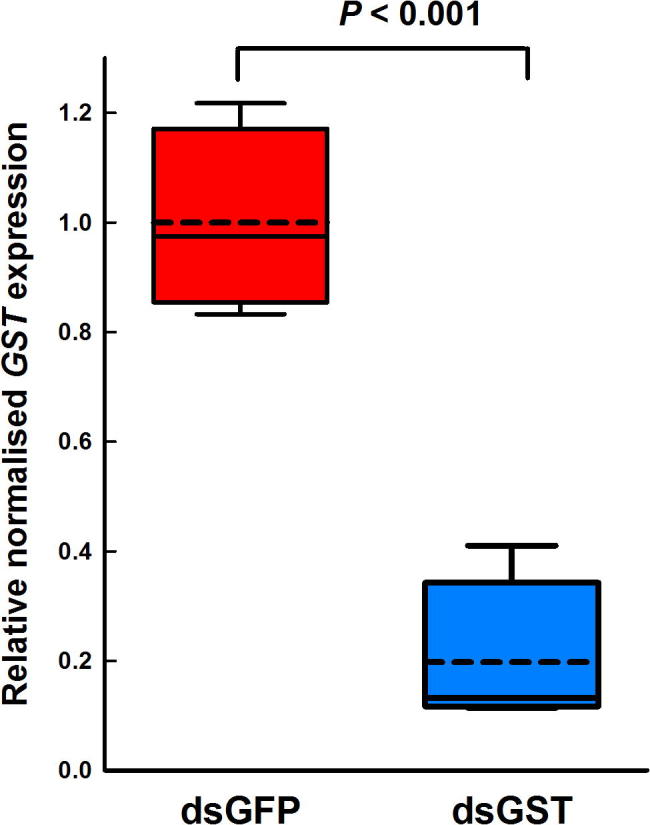
Relative normalised expression of *GSTσ* 96 h in *Ctenocephalides felis* after injection of double-stranded *GFP* (ds*GFP*) (*n* = 4) or ds*GSTσ* (*n* = 4). *GSTσ* expression is significantly lowered (*P* < 0.001) in the ds*GSTσ* – treated groups. Boxes indicate the interquartile ranges, whiskers the 10th and 90th percentiles, internal solid line indicates the median and dashed line indicates the mean. All data are normalised using two reference genes and expressed relative to the mean expression of the gene of interest in the ds*GFP* control.

When groups of adult *C. felis* were injected with ds*GSTσ*, the expression levels of *GSTσ* decreased by 80% compared with those seen in ds*GFP* injected controls (0.2 ± 0.07 versus 1 ± 0.08, *P* < 0.001 mean relative normalised expression ± S.E.M.) (Fig. 1). This indicated that *C. felis* are capable of mounting an RNAi response, at least when exposed to exogenous dsRNA delivered directly into the haemocoel and bypassing the gut barrier.

### 3.2 Survival of *C. felis* adults after microinjection or immersion

**Fig. 2:**
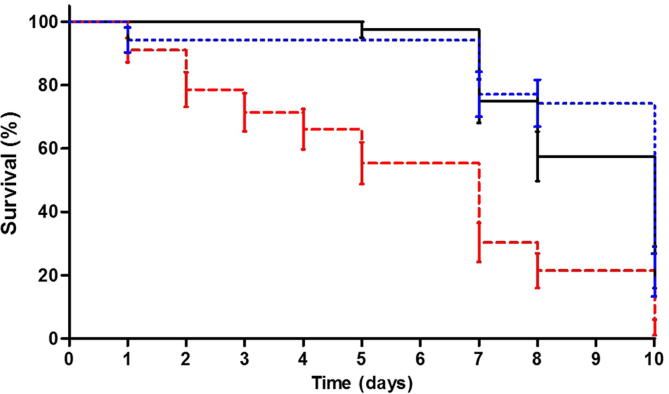
Kaplan-Meier survival curves for *Ctenocephalides felis* adults after no treatment (*n* = 40) (solid line), soaking (*n* = 35) (small dashed line) or microinjection (*n* = 56) (large dashed line) of saline. Surviving fleas were counted at intervals for 10 days post-treatment. Error bars show S.E.M.

There appeared to be an increase in *C. felis* mortality in the first trial following the invasive microinjection procedure. To assess the impact of this delivery method on mortality, adult *C. felis* were either injected with 0.9% saline, immersed overnight in 0.9% saline or not treated, and their survival was monitored for the following 10 days. A Mantel-Cox test demonstrated that survival differed significantly (*P* < 0.05) between the three treatments (Fig. 2). Survival curves indicated there was decreased survival of cat fleas following microinjection (Fig. 2).

### 3.3 Immersion in dsRNA causes specific target and transient gene knockdown in *C. felis*

**Fig. 3:**
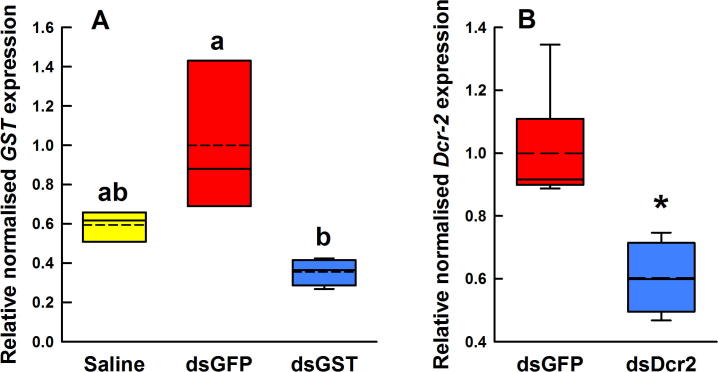
Relative normalised expression of target genes in *Ctenocephalides felis* adults after immersion in double-stranded RNA (dsRNA) solutions or saline. All values were normalised to two reference genes and presented relative to the mean normalised expression level of the ds*GFP* treatment. (A) Relative normalised expression of *GSTσ* following immersion in ds*GSTσ* (*n* = 4), saline (*n* = 3) or ds*GFP* (*n* = 3). Treatments not sharing the same letter are significantly different (*P* < 0.05). (B) Relative normalised expression of *Dicer-2* after immersion in ds*Dicer-2* (*n* = 4) or ds*GFP* (*n* = 6). ^*^P = 0.004 which is statistically significant.

The *GSTσ* expression level was significantly decreased by 65% in the ds*GSTσ*-treated fleas relative to the control ds*GFP*-treated fleas (0.36 ± 0.03 versus 1 ± 0.22, *P* < 0.05) 72 h after administration of the dsRNA by immersion. Similarly, immersion in ds*Dicer-2* caused *Dicer-2* mRNA levels to decrease to 0.6× (*P* = 0.004) the level in the ds*GFP*-treated control groups (Fig. 3B) 48 h post-treatment.

**Fig. 4:**
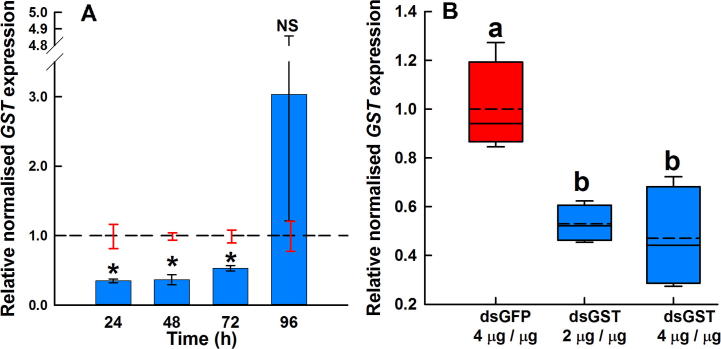
Duration and dose-dependence of double-stranded RNA (ds*GSTσ*) expression in adult *Ctenocephalides felis* following immersion in dsRNA. (A) Relative normalised *GSTσ* after immersion in 2 µg/µL of dsRNA (bars, mean ± S.E.M.) relative to the mean *GST*σ level in control groups (ds*GFP*–treated, dashed line, mean ± S.E.M.) at the given time point. ^*^P < 0.05; NS, not significant compared with the control group at similar time point. *n* = 3 or 4 groups of 10 fleas. (B) Relative normalised *GSTσ* expression in fleas 72 h post-treatment by immersion in 2 µg/µL of ds*GFP*, 2 µg/µL of ds*GSTσ* or 4 µg/µL of ds*GSTσ*. *n* = 4 groups of 10 fleas. Treatments not sharing the same letter are significantly different (*P* < 0.05).

Groups of fleas were immersed overnight in ds*GSTσ* or ds*GFP* dsRNA and samples collected 24, 48, 72 and 96 h after removal from the solution. At time points 24–72 h after removal from dsRNA solutions, the levels of *GSTσ* were significantly decreased in ds*GSTσ*-treated groups relative to the ds*GFP*-treated control groups at the same time points (24 h, 0.35 ± 0.03, *P* = 0.021; 48 h, 0.36 ± 0.07, *P* = 0.002; 72 h, 0.53 ± 0.04, *P* = 0.003) (Fig. 4A). Although ds*GSTσ* treatment caused significant knockdown relative to ds*GFP*-treated controls, by 47–65% 24–72 h post-treatment, there was no significant difference in the degree of knockdown across these time points (*P* > 0.05). By 96 h post-administration of the dsRNA there was no significant difference in *GSTσ* levels between the treatment and control groups (Fig. 4A).

The effect of dsRNA dosage on the extent of knockdown was tested by immersing groups of *C. felis* in either 2 μg/μL of ds*GFP*, 2 μg/μL of ds*GSTσ* or 4 μg/μL of ds*GSTσ* and assessing *GSTσ* expression 72 h post-treatment. The relative normalised *GSTσ* level was significantly lower (*P* < 0.05) in both the 2 μg/μL (0.53 ± 0.04) and 4 μg/μL (0.47 ± 0.10) ds*GSTσ* soaked groups compared with the ds*GFP* control groups (1 ± 0.09) (Fig. 4B). However, there was no significant (*P* > 0.05) difference in *GSTσ* levels between the two ds*GSTσ* dosages tested.

### 3.4 Feeding dsRNA triggers significant knockdown of a target mRNA in *C. felis*

**Fig. 5:**
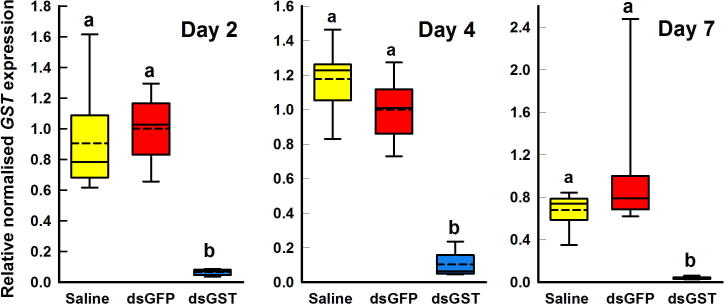
Relative normalised expression of *GSTσ* in adult *Ctenocephalides felis* following prolonged feeding of double-stranded RNA (dsRNA) or saline in blood. All values are expressed relative to the average normalised expression of the ds*GFP* control and normalised using two reference genes. *n* = 8 groups of 15 fleas. Treatments not sharing the same letter are significantly different (*P* < 0.05).

To assess if feeding dsRNA within a blood meal could trigger knockdown in *C. felis*, adult *C. felis* were continuously fed either a blood-saline mix, or a mixture of blood and ds*GSTσ* or ds*GFP* (0.11 μg/μL). Prolonged and continuous intake of ds*GFP* had no significant effect (*P* > 0.05) on expression levels of *GSTσ* (Fig. 5A–C). In contrast, within 2 days (Fig. 5A) and sustained up to 7 days (Fig. 5C) of continuous feeding of ds*GSTσ*, there was significant (*P* < 0.01) and robust (up to 96%) knockdown of *GSTσ* mRNA in ds*GSTσ* fed fleas, relative to both the ds*GFP* and saline controls (Fig. 5). In ds*GSTσ*-treated fleas *GSTσ* levels were 0.07 ± 0.01, 0.10 ± 0.03 and 0.04 ± 0.004 relative to ds*GFP*-treated fleas (1 ± 0.08, 1 ± 0.06 and 1 ± 0.22), at days 2, 4 and 7, respectively.

### 3.5 Upregulation of siRNAi machinery following exposure to dsRNA

**Fig. 6:**
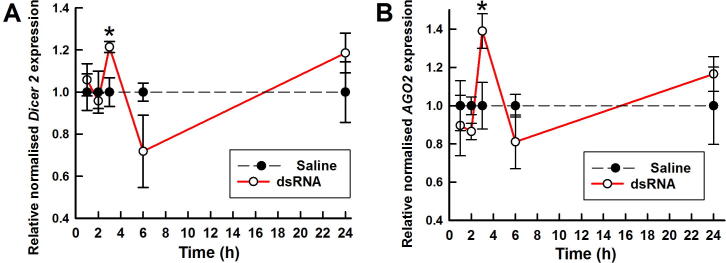
Expression of *Dicer-2* and *AGO2* in *Ctenocephalides felis* 48 h after exposure to overnight immersion in 2 µg/µl of double-stranded *GST* (ds*GST*) (solid lines) or 0.9% saline (dotted lines). All values are displayed as the mean normalised expression ± S.E.M. of the gene of interest relative to the saline control at that time point. (A) Relative normalised expression of *Dicer-2*. (B) Relative normalised expression of *AGO2*. *n* = 4 groups of 10 fleas. ^*^P < 0.05 (statistically significant difference from saline control at a given time point).

To investigate whether siRNA RNAi machinery is upregulated in response to dsRNA, groups of *C. felis* adults were exposed to dsRNA by soaking overnight, then sampled at set time points after removal from dsRNA solution and the expression of *Dicer-2* and *AGO2* assessed. *Dicer-2* and *AGO2* were both significantly, but only marginally, upregulated 3 h after exposure to ds*GSTσ* (Fig. 6A and B). *Dicer-2* expression was increased to 1.2× (*P* = 0.026) and *AGO2* expression was increased to 1.4× (*P* = 0.042) at 3 h, relative to cat fleas which had been exposed to saline alone. No significant differences in *Dicer-2* or *AGO2* expression levels, relative to saline controls at equivalent time points, were observed at the other time points tested (1, 2, 6 and 24 h post-exposure).

## 4 Discussion

Gene knockdown by administration of dsRNA to induce RNAi is a powerful technique allowing researchers to investigate the physiology of pests and parasites, and to identify potential novel drug targets. Indeed, RNAi itself has often been stated as a potential pest control agent and now there is increasing confidence that dsRNA-containing pesticides may reach the market within a short number of years, as costs of dsRNA production fall to US $2 per gram and regulatory approvals are being granted (Zotti et al., 2018). This study demonstrates RNAi in the cat flea for the first known time and provides insights into the dynamics of the response of *C. felis* to exogenous dsRNA administered by various routes.

Assuming that a species has the necessary machinery to execute RNAi, many studies have demonstrated that haemolymph dsRNA concentration is a key factor for RNAi efficacy. The concentration of dsRNA in haemolymph can vary due to degradation of dsRNA by endonucleases in the haemolymph if dsRNA is delivered by injection or in the midgut juice if dsRNA is ingested (Wang et al., 2016, Singh et al., 2017). Gut nucleases are particularly active in some species, with RNAi efficiency reported to be poor, or absent altogether, in several species when the dsRNA is administered by ingestion rather than injection (Luo et al., 2013, Garcia et al., 2017, Song et al., 2017). To overcome this possible barrier, intrahaemocoelic microinjection was selected as the initial route of administration, as it is the most direct method, delivering dsRNA directly to the haemocoel and bypassing potential gut nucleases. Injection of ds*GSTσ* into *C. felis* was able to knockdown the target gene by approximately 80%. This was the first known demonstration of specific knockdown of a target gene in response to exogenous dsRNA in the cat flea or any other Siphonapteran. While microinjection of dsRNA could knockdown the target gene, the technique is labour-intensive, requires specialised equipment, and is highly invasive with decreased survival of *C. felis*.

Immersing whole fleas in dsRNA was investigated as a less invasive administration method, having been used successfully in a number of small arthropods such as *Aedes aegypti* larvae (Singh et al., 2013, Bona et al., 2016) and several mite species including the honey bee mite *Varroa destructor* (Campbell et al., 2010), the house dust mite, *Dermatophagoides pteronyssinus* (Marr et al., 2015), and *Sarcoptes scabiei*, the scabies mite (Fernando et al., 2017). In *C. felis*, soaking in dsRNA was able to significantly knockdown *GSTσ* by 65% and *Dicer-2* by 40%, which is comparable to knockdown reported for *D. pteronyssinus* (Marr et al., 2015) and higher than for *S. scabiei*, the scabies mite (Fernando et al., 2017). The degree of knockdown we observed in *C. felis* was lower than reported for *V. destructor* (Campbell et al., 2010, Campbell et al., 2016) although similar approaches were taken. No increased level of knockdown was observed when the fleas were immersed in double the concentration of ds*GSTσ*, indicating that the limiting factor was unlikely to be the amount of circulating haemolymph dsRNA. Assessing the degree of knockdown of *GSTσ* across several time points indicated knockdown was highest 24–48 h post-immersion in ds*GSTσ*, but never above 65% knockdown.

Administering dsRNA by ingestion has proved to be the most problematic delivery method in other arthropod species, likely due to the action of dsRNA degrading nucleases secreted into the gut lumen (Luo et al., 2013). In some species, the gut nucleases themselves need to be suppressed by gene knockdown before the target gene can be efficiently knocked down (Garcia et al., 2017, Song et al., 2017). Unexpectedly, however, feeding *C. felis* ds*GSTσ* in a blood meal was extremely effective in decreasing levels of the target gene by up to 96% and observed within 2 days of feeding. There are very few reports of dsRNA being administered by ingestion to haematophagous arthropods: knockdown of *isac* in *Ixodes scapularis* fed dsRNA in buffer (Soares et al., 2005); *nitrophorin-2* in *Rhodnius prolixus* by 42% when fed dsRNA in media (Araujo et al., 2006); and *V-ATPase* by 60% in *A. aegypti* fed dsRNA in sucrose solution (Coy et al., 2012). Indeed, we are aware of only two reports of dsRNA administered in whole blood: knockdown of *TsetseEP* by ≤58% in *Tsetse morsitans morsitans* (Walshe et al., 2009), and knockdown of *ubiquitin* in *Rhipicephalus microplus* (Lew-Tabor et al., 2014). It is noted knockdown efficiency in *T. m. morsitans* was consistently lower when dsRNA was administered by ingestion rather than injection (Walshe et al., 2009). It is unclear why such superior knockdown in *C. felis* was achieved when the ds*GST* was administered by continuous feeding compared with the injection or soaking routes, but it appears that gut nucleases are not an impairment to RNAi in *C. felis*, unlike in many other species. Further, our results indicate that dsRNA is stable in whole blood and blood is a suitable medium for feeding dsRNA to haematophagous arthropods over extended periods.

Artificial feeding methods are used commonly in rearing of cat fleas, but also for compound testing (Shoop et al., 2014, Kernif et al., 2015, McTier et al., 2016). Feeding offers a non-invasive way to continuously expose *C. felis* to dsRNAs and the use of pre-existing equipment makes it promising for various applications. Therefore, dsRNA feeding could potentially be employed for higher throughput RNAi screens for the identification of knockdown-associated phenotypes and potentially as a drug target validation method in *C. felis*.

To confirm their roles in siRNAi in *C. felis*, the expression of *AGO2* and *Dicer-2* was assessed following exposure to exogenous dsRNA. The increased expression at 3 h after dsRNA exposure was modest for both *Dicer-2* (1.2×) and *AGO2* (1.4×) relative to upregulation of siRNAi machinery seen in some other insects. For example, after exposure to dsRNA *Dicer-2* was upregulated approximately 5× in *Blattella germanica* (Lozano et al., 2012), ≤28× in *L. decemlineata* (Guo et al., 2015) and ≤360× in *Manduca sexta* (Garbutt and Reynolds, 2012). A possible reason for the low level upregulation observed in the present study of *C. felis* is that the dsRNA was introduced by soaking, rather than injection. Notwithstanding the modest magnitude of the response, upregulation of *Dicer-2* and *AGO2* in *C. felis* following exposure of dsRNA implicates the siRNA pathway in the RNAi response observed.

Until this study, RNAi had not been demonstrated in *C. felis*, or other siphonapterans. It has now been successfully shown that *C. felis* can mount a strong RNAi response upon exposure to dsRNA through a variety of delivery methods. While each method has its advantages and drawbacks, delivery of dsRNA within a blood meal would be recommended for future studies, due to the efficacy, ease of delivery and sustained effect. However, soaking in dsRNA also offers a simple, rapid, non-invasive approach which can effectively knock down target genes to a degree comparable, or better, than many other species. An unexpected finding was that RNAi was more effective when dsRNA was delivered by feeding rather than injection, suggesting that *C. felis* has lower gut nuclease activity than other studied species. This study represents the first step in utilising RNAi in the cat flea, opening up new avenues for research into this important pest.
