# Supplementary material for: RNA interference in the cat flea, Ctenocephalides felis: Approaches for sustained gene knockdown and evidence of involvement of Dicer-2 and Argonaute2
Source: Int J Parasitol. 2018 Nov;48(13):993–1002. doi: 10.1016/j.ijpara.2018.04.006 (PMC6237673; doi:10.1016/j.ijpara.2018.04.006)
Supplement: Supplementary Data 1 [file mmc1.docx]

**Supplementary Data S1.**

Sequences for *Dicer2* and *Argo2* genes were identified in a newly assembled in-house *Ctenocephalides felis* transcriptome using BLASTx, E-value cut off 1e^-20^, searching with protein sequences retrieved from GenBank (*Bombyx mori* *Dicer2* (**AB566386.1**); *B. mori Argo2* (**NM_001043530.2**)). The sequences used for subsequent primer design are shown below, with primer sequences indicated.

Dicer 2 Locus_1_Transcript_32157/167557

TGGGAACAATATTCGGTGAATTCCCCATTTATCTCGTTCTGTGTGCGCTTAAAGTGATATACTTTGATTAGTTCATTTTTATAGAAAGTTTATTTTAGTGATATATTAGTGTGATTAGTGTTAGAAACGGATTAGTGAATATTTTAATAACTGCGATATTTCAATTTTAATGACATGGATGATCAGGACTTTCGAGCTCGTCCATACCAAGTGGACCTGTTGCAAATTGCTATACAAAGAAATACGATTGTATTTTTACCTACCGGGTCTGGGAAAACGTTTATCGCATTGCAAATGCTGAAATATTTATCTAGTGAATTGGAAAAGAGTCTTGACAGTGGAGGTAAGAGAAGTATATTTTTAGTAAATACAGTGCCTCTCGTTGAGCAGCAAGCAAAATTTATTAAAAGCCAGACGTTTATAAAGAATGTTGGGGAATATACTGGTGCAAAAAATGTGGATAATTGGACTAGAGAAAAATGGCTCAATGAGTTTAACCAGCATAAGGTACTCGTGATGACAACTCAAATATTAGTTGATGTACTCAATCATGGTTATTTGTCATTAAGTAATATAAATTTAATTATAATGGATGAATGTCATCATACAATAAAAGAACATCCAATGCGTCAGCTCATGTTGTTTTATAAGAATCGTAGAGATGAAAACATAAGCTTACGAATTTTGGGGTTGACAGCATCTTTAATAAATTCAAATTGTAAACCTCATAACGTGGAAATAGAAATAAATACTTTAGAAACCTCAATGGGATCCACTATAGCAACTATTGATAATATGGAAAGTGTATTGGGATTTTCAGCAAATCCAAGAGAACGAATAGTATTTTATGATAATCAAAAAACACCACAAATAATAACAGATTTGATAAGTGAATTGAGTGTTACTACAGACAAATTTGCCATATTACAACATGGGAAAGTTATTCCAGAGCATGAATCTATGGTGTACATGAGAGGAGGATGTAAAGATGCTCGAAAAACAGTATGCAATATGATTAGAGATTTTATTGAAGATATGATAACCCTGGGGCCATTTGGTGCCCATGAATCTGTTAAAATTCGTCATTTAGAAGTAGAACAAATGAAGAGGGTAGCATCAAACAGACAAGATGCTATGTTATATTCTCAATTAATTACTGTGTTTATGTATGTAAAAAAAAAATTAAGAGCGTATATGGAGTCACAAGGTGCTGTAGAACAGATTTATCAAAATTCTACTCCCAAAGTATTGAAATTAATTGAGGTTATTAAAGAGTATTCTCCTAAACAAAATAATAAGAAGCTTCCCCTAAAGGCAATAATTTTTGTACAAAGGAGATTTACGGCAAATATGCTATATCAAATTTTCAAAGCATTATCTAGAGAGTGCAACGATTTTAATTTTATTCAACCAGAATTTGTAATTGGTATGTCCAACAGAGGAATGATTCAGGGAATAGAGAGTGCTTTGGATCGTAAATGGTGCAAAAAAGCACTAATCAAATTCAGATATGGAGATGCAAATTTGCTAATTGCATCAAATGTATTGGAAGAAGGTATAGATGTGCCAGAGTGTTCACTTGTTGTGAGATTTGATTCCATTCAGAATTATTGTTCATATACACAATCTAAAGGCCGAGCAAGAAGCAAGGATAGTGAATTTATAACATTAGTTTGTGCTGATGACAAAGACAAATTTATGACAAAATATTATCAATTTAAAGAAGTTGAGAAAAATTTATTGCATTTGCTGGTTGGCAAAACCAATGAAAGAATTGCGCCTTCTCTAGATGATTTTGAGGATCCAATTATTCCTCCTTATGAAACAGATCAGGCACAAGCAACACTTTTATCTGCAATTTCTCTAGTCAATCGTTACTGTTCTGCACTTCCTCAGGATATGTTTACTCATCTTACAGTCAATTACTTTCGAGAACATAAGGTTATAGGTAATTTTACTAGATATCGTGTGATGTTACAGTTTCCGATTATGTGCAAGCTACGTGAAGGAATTATGAGTGATTGGATGACTTCTATAAAAAATGCCAAGCGACATGCAGCCTTATGTGCGGTTAAACTTCTCCACGAAACTGGAGAATTAGATGACCATTTGATGCCCATAACTAATCGCAAACTCAATGAAATGAAGGAGTTGCATGGTTACTTTGACCATTGGTTAAGAAGTGAACCAACCCACGATGCACAAGGAAATTTTCTCAGTAAACCTGGAACAAGAGGACGATTCAGACTTCATAACAGAATCATACCACTAGATTTGAAACGTTGTGGGCCTGTTGCTGACAATGATGTGTATTTACATGTTCTTGAAATATCTGCAGACTACAGCAAACCATCAGAGTTAGATAACAGAGCTTTGAAGTTTTATAATATGCTCAATTCCAAACAAACTTATGCTTTTCTATCTTCTAAACCATTTCCTTATTTGTGTGATTTTCCACTCTTCCTGAGCGTTGGAAGATTGCATGTTAAGCTCCGTATGAATCATGGCTTCTTGAGTTTAACAAATAATGACATAAAAAGGATAAGATTGTTTAATGTGATGCTCTTTAAAGATGTCTTACAAGTATTAAAAAGTTTTTTGATTTGTGATACTTTAGATGAAGAAGTGTCATTATTGGTTTGTCCAGTTGATACAAATTGGAATATAGATTGGCAAATTATAGACAGTTTTCCTGAATTAATAATACCGGATGCTCCTACACACCAACAAAGAAAGGAAATGGTTATAGATCCTGAAAATTACAGAGATGCCATTGTTGTACCTTGGTACAGAAATATTACTTCTAAAACTATATACATTGTTACTAGAGTGTGTGAAGAAATGTCTCCCCTCTCGAATTTTCCAACTGAATCTTATGATACATACAATTCATATTTTCGAGACAAATATCACTTAGAAGCCTGTCATATGAAGCAACCTCTCTTAGAAGTTAAACCATTTTCTTTTAATAAATTGAACTGTCTCAAACCAAGGGCTAGTAAAAGCAAGAAATATTTCAATATTGACTTGGATAGTTCTGCATTCGATGAGCACCTTGTGCCTGAATTCTGTGTGATAATAAAATATCCTGCAATTTTGTACCTAAAATCGGCTTTATTACCCAGTATTTTTCATCGTTTTTTATCTATGATGAGAGCCCGGGAGTTAATTAATATTATTAATACAGAATTAAATCTCTCTGAACCATATTTTAAAACCTCTCCAATTGGATGTCGGATAGAACTAATGCATGATCCGGAAGAGCCAGATTCTTCAAGTAAATGTAACAACCAAGCAGTTAATTTGGACCAAGCCAGTGATAGTAGTTCTAATTATAGTCATGATTCAGACATTATGGATTTTTTACCGAAATTAGATAGTAAAGATCTTACTAACTATGATTTTGAAATTCAAAAATTAAACAAAGAATATGATTGGGATGTTTCTATTGAACCGGTAGATTTTCATCGCAATGCTGATGGTACATTGAAAGTTGACATGGATTACTATTACAAATTTGTAAAAAATAAAATTGATGATAATATTGAAACTGAGAATAATGTTCCTTTAGGAATTGATCATCAACAATTGGCTATTTGTTATGAAGATACTGTAATTCCTTTAGCAATGTTAAATGAGCACTCACAAATTGTGGAATTGAAGGATATGTTAGCTGTTCTAACAGCTAAAACAGCGAATGATTTATTTGATTTGGAACGTCTAGAAACTTTGGGTGATAGCTTTTTAAAATTTGCTGTAACCTTATATCTTGCTGACAAATTTCGAGATTTGAACGAAGGTCATCTGACAAGTTTTAAAGGACAGATTATAGGGAACAAAAATTTAATGTATTGCGGTTTGAATAAGCATTTGGCTGGTTATATGAATATCTATGAATTTGCTCCTTGTGCTGATTGGATACCACCTGGGTTTACCCTCTCCAAAGATCTTAAAACAAATTTGATTAATAGAGATGTATCAGCAGAAATGTTATACGGACAACTTGTACCAAAGGAAGAAGTGCTATCTGGGGTATTATCTCCTCAAACTTTGAAAGCAATGCTCACCGCGCTGAACGAAATATGCGAGGACTCTACTGAATCAAATACTAATACGTTGCAAAATTTAGTTGGAATGTTAAATGTTTCTGATAAGACCATAGCCGATTGTATTGAAGCTTTAATAGGTCAATATCTTTTGTCTCACGGTGTTAATACATGTTTATCATTATTGGCGTTTTTGAAAATTTTGCCTTACTCGGATGAATTAGTCCATCTCATGGAAAGCGAAGCGATTCCTGCTAAATTAAGCAATGAAATTACGGAAAGTGATATAGATTATTTCATACCGGGTTACGCAACTTTGGAAAAATCTTTAGGTTATCAGTTTAAAGATCGGTCTTATTTACTTCAGGCTCTGACCCACGCATCTTATTCTATGAACCGGCTCACTGACTGTTACCAGCGTTTAGAGTTTGTCGGAGATTCTATATTAGATTTCTTGATAACAGGTCATATATATGAGCATTGCGGTCTATTATCGCCTGGTGAGCTTACTGATTTACGCTCAGCCCTAGTCAATAATAACACATTTGCAAGTCTTACAGTCCGATATGGATTTCACGAGCACATATTATCGTTAAGCCCCAGTCTGGCCGATTCAATTAATAAGTTTGTTTCATTTCAAGAACAGAGAGGACACAAAGTAGATTTGGAACTACTTCATCTTTTGGAAGAGGGAGAATGCTCCGTAGTGGAGAGTGTCGACGTTCCAAAAGTTCTAGGGGATATTTTCGAGTCTCTTGCCTGTGCCGTTTTCTTTGACAGCGGTAAAAATTTGAATGTTGTTTGGAACGTATTCTCTAAATTGATGAATAATGAAATCAAAATGTTCTCGAAAAATGTACCGAAACAAATTGTTAGGCAATTATATGAGACAAATTGTAAGCCAAAGTTTGGGGACACTCTATTACCCGATAAATCTAAATGTGTAGCAATTTCTTTGACATTTAGTCACGAAGGAAAACTGCACACCGTTTATGGCGTCGGAGTAAATAAACAGAACGCGAAAAAAGCTGCTTCTAAAATGGCTCTTCAAATGATGGGTCGTCATTAAGTACTATAAGTACCAAAATGGCGTAAAGTATAAATATTTATTAAAATTGATAAATAAATATTTATACAATAAA

Primers sequences (highlighted)

dsDicer2 F CAGTTCCTCGAGATCGCAATGCTGATGGTACA

dsDicer2 R GCAGCAGATCTCAGCACAAGGAGCAAATTCA

qDicer2 F CATCTCATGGAAAGCGAAGC

qDicer2 R AAGTTGCGTAACCCGGTATG

Argonaute 2 Locus_1_Transcript_26661/167557

CTCCTCCCCCGGGCAGCACAGTAGGCCCTAGTAGTGGTGTGACACAAACAGGTTCTGCAACTGTTCCGACGGGATCTTTAGGCCTTGTGCCTGCCAGTGCAACACATCAACCACCCGCACCTCCAGAATTACCAGTATTTTCATGTCCACGTCGACCAAATTTGGGCCGCGAGGGCAGGCCAATTGGTCTTCGGGCTAATCATTTTCAAATAACTATGCCAAGGGGATTTGTACATCACTATGATATTAATATACAGCCAGACAAATGTCCAAGGAAGGTGAATAGAGAAATCATAGAAACTATGGTGCATGCATACAGTAAAATATTTGGAACTTTAAAACCTGTATTTGATGGCCGAAATAATTTGTACACGAGGGATCCTCTTCCAATTGGCAATGATAGAGTGGAATTGGAGGTGACACTACCTGGGGAAGGAAAGGATAGAGTTTTTAGAGTTACCATAAAATGGGTGGCACAGGTGTCTTTGTATGCTTTAGAAGAAGCACTAGAAGGACGCACTAGACAAATTCCATATGATGCTATTTTAGCATTGGATGTAGTTATGAGACATTTACCTTCAATGACATACACACCAGTTGGAAGAAGTTTTTTTAGCTCACCCGATGGTTATTACCATCCTCTGGGTGGTGGCCGTGAAGTTTGGTTTGGTTTTCATCAAAGTGTTCGACCTTCACAGTGGAAAATGATGCTTAATATTGATGTATCTGCAACAGCTTTTTATAAGGCTCAGCCTGTCATTGAGTTCATGTGTGAAGTTCTTGACATAAGAGACATTAATGAACAAAGAAAGCCTTTAACAGATTCTCAGAGAGTTAAATTTACAAAAGAAATTAAAGGGCTTAAAATTGAAATTACTCATTGTGGCACAATGAGACGCAAATATCGTGTTTGTAATGTAACTAGAAGGCCAGCACAAATGCAATCATTTCCACTGCAATTAGAAAATGGACAAACAGTAGAATGTACTGTAGCAAAATATTTCTTAGACAAGTATAAAATGAAACTACGCTACCCACACCTGCCTTGCTTGCAAGTCGGACAAGAACACAAACATACATATTTACCACTGGAGGTATGCAATATTGTAGCAGGGCAACGATGTATCAAGAAATTAACTGATATGCAGACATCCACAATGATTAAAGCAACTGCCCGGAGTGCACCTGATAGAGAGAGGGAAATCAATAATTTGGTGCGTCGAGCAGATTTCAATAACGATTCTTATGTTCAAGAATTTGGTTTAACAATAAGCAATAACATGATGGAAGTTCGAGGGCGTGTTTTACCACCACCAAAACTGCAATATGGGGGCAGAGTTTCACTTCTAAGTGGCCAAATGGTACCTTGTTCACAGAGTAAACAACAAGCACTGCCTAATCAAGGAGTGTGGGCTATGAGAGGAAAGCAGTTTTTTACGGGTGTTGAAATACGTGTGTGGGCCATTGCCTGCTTTGCCCCTCAGAGAACAGTTCGAGAAGATGCTTTAAGAAATTTTACACAACAATTACAGAAAATATCAAATGATGCTGGTATGCCTATTATAGGACAACCGTGTTTCTGTAAATATGCAACAGGGCCTGATCAAGTTGAACCTATGTTTAGGTATCTTAAATCTACATTTTCGGCTTTACAATTAGTTGTTGTAGTTTTGCCAGGAAAGACACCAGTTTATGCTGAAGTGAAACGAGTTGGTGATACAGTTTTGGGAATGGCAACTCAATGTGTTCAAGCTAAAAATGTAAATAAAACATCTCCACAAACATTAAGTAATTTATGTCTGAAAATTAATGTTAAATTGGGTGGTATAAATTCTATTTTAGTTCCATCTCTCAGACCAAAGGTATTTAATGAACCAGTTATATTTTTGGGTGCGGATGTAACTCATCCACCTGCTGGAGATAATAAAAAACCATCAATAGCTGCTGTAGTAGGCTCTATGGATGGGCATCCATCTCGTTATGCAGCTACTGTTCGAGTTCAACAACATCGTCAAGAAATTATTCAAGAACTTAGTTCGATGGTTAGGGAACTGCTGATCATGTTCTATAAAAGTACTGCAGGATATAAGCCACATAGAATTATATTATACAGAGATGGAGTATCAGAGGGCCAATTTTTGCATGTTTTACAACATGAATTGACGGCTATTCGTGAAGCTTGTATTAAATTAGAATCAGAATACAAACCTGGAATAACATTTATTGTTGTGCAAAAAAGGCATCACACTAGACTTTTTTGCTCTGATAAGAAAGAACAATCAGGGAAGTCAGGTAATATACCAGCTGGTACCACAGTTGATGTTGGCATTACCCATCCAACAGAATTTGATTTTTATCTATGCAGTCATCAAGGCATTCAGGGTACTAGTCGTCCCTCACATTATCATGTATTATGGGATGATAATCATTTTGATTCTGATGAGCTGCAGTGTCTTACCTACCAACTATGCCACACTTACGTTAGATGTACACGTAGTGTTTCAATACCTGCCCCAGCATATTATGCTCATCTAGTAGCATTTAGAGCTAGGTATCATTTAGTGGAAAAGGAGCATGACTCTGGAGAAGGATCACATCAGTCTGGATGCTCTGAGGATCGTACACCTGGCGCTATGGCAAGAGCAATAACAGTACATGCCGACACCAAGAAAGTAATGTATTTTGCTTAATTCAGGTAAGTATGTTTATAGGAATCACACTTAATTTTATTGAGTAGTTATTAGTCAGTATCCAGTTTTGAATACTAAACTTTTACATGCTGATTTGAATTATTTATTTGATAGATTTTATTCAAATGCAAATTATAATTGCAACATGATTTTTTTTTAAGAACTTTTATTATGTTTGACAATTAAAAGAAAAAAATTGTTTTAAAAGTTTAGTTTATTTTATATTGAAATTTTGCTTTAATTTTGACACTTTTATTTGGAATTTCCAAGTCTTTTTTTCCTGTTCGCATCACAACAAAGTTGAAATCATTCATGTTATTTTCACA

Primers sequences (highlighted)

qArgo2 F CAATATGGGGGCAGAGTTTC

qArgo2 R ATGGCCCACACACGTATTTC
